# Factors Related to Suicidal Ideation and Prediction of High-Risk Groups among Youngest-Old Adults in South Korea

**DOI:** 10.3390/ijerph191610028

**Published:** 2022-08-14

**Authors:** Eungyung Kim, Jee-Seon Yi

**Affiliations:** 1 Department of Nursing, Chungbuk National University, Cheongju 28644, Korea; 2 College of Nursing, Gyeongsang National University, Jinju 52727, Korea; 3 Institute of Health Sciences, Gyeongsang National University, Jinju 52727, Korea

**Keywords:** suicidal ideation, old age, decision tree analysis, high-risk, depression, personal income, perceived health

## Abstract

(1) Background: The suicide of older adults shows different factors between the youngest-old adults and the old-old adults. This study aimed to identify factors predicting suicidal ideation among youngest-old adults (ages 65 to 74 years) and predict high-risk groups’ characteristics. (2) Methods: The subjects of this study were 970 youngest-old adults who participated in the Korean National Health and Nutrition Examination Survey (KNHANES VIII Year 1, 2019). Logistic regression analysis identified factors related to suicidal ideation, and decision tree analysis identified combined characteristics among high-risk groups. Data were analyzed using SPSS 27.0. (3) Results: Suicidal ideation became more common among those with relatively lower income levels (OR = 1.48, 95% CI = 1.04–2.12), those whom had experienced depression (OR = 9.28, 95% CI = 4.57–18.84), those with relatively higher stress levels (OR = 2.42, 95% CI = 1.11–5.28), and those reporting a relatively worse perceived health (OR = 1.88, 95% CI = 1.23–3.11). Complex characteristics that combined depression, low personal income level, and low perceived health predicted a high risk of suicidal ideation (64.6%, *p* < 0.05). (4) Conclusions: The findings indicate that this high-risk group should be prioritized when developing suicide prevention strategies.

## 1. Introduction

Old age may limit physical and mental abilities, affect social and economic activities, and be accompanied by chronic disorders [1]. These characteristics may threaten older adults’ quality of life and possibly trigger depression and even suicidal ideation [2]. Although suicide rates vary from country to country, suicide among older adults is a global public health issue, as they commit suicide at higher rates than other age groups [3,4]. Notably, South Korea’s suicide rate has ranked high among Organization for Economic Co-operation and Development (OECD) countries for over ten years [5,6]. In 2020, the suicide rate among older adults was 41.7 per 100,000 people in South Korea [7]. Under these circumstances, suicide among older adults in South Korea requires serious attention, especially considering that the country’s aging rate is the fastest in the world [5].

Suicide is directly related to suicide attempts, which occur in the reciprocal process of various risk factors, including socioeconomic, biological, and psychological factors [8]; those who have contemplated suicide are at higher risk for suicide attempts compared to those who have not [9]. Suicidal ideation is the most salient short-term warning sign preceding suicidal attempts [10,11]. Furthermore, older adults commit suicide without expressing their suicidal intent following long-term experiences of suicidal ideation, and the success rate of these suicidal attempts is high [11,12]. Jeon [13] found that 98.5% of older adults who had attempted suicide had also experienced suicidal ideation, indicating a more deliberate tendency than other age groups. Thus, the optimal strategy for reducing suicide attempts should identify risk factors for suicidal ideation and intervene based on these factors [14,15].

Previous studies [16,17,18] with older adults have reported that their suicidal ideation is related to physical factors (e.g., various physical changes and disease morbidities due to aging) and social factors (e.g., social isolation and lack of support), economic hardship, or psychological factors (e.g., depression). According to a recent study, reasons why older adults consider suicide include diseases and disabilities (37.5%), economic hardship (28.1%), loneliness and social isolation (15.9%), and family conflicts (11.0%) [19]. Additionally, older adults occasionally suffer from comorbidities of various diseases, such as stroke, heart disease, and chronic lung disease, and depression is highly likely to accompany the deterioration of individuals’ physical health [20,21]. Furthermore, depression is the most common mental health issue connected to suicidal ideation [11,21,22]. A survey [23] revealed that 21.1% of older adults in South Korea were experiencing depression, 6.7% had thoughts about suicide, and 13.2% had attempted suicide. Moreover, the absence of a spouse, living alone, being male, and loneliness are predictors of suicidal ideation among older adults [24,25,26]. Because suicidal ideation among older adults is related to various complex factors [12], research should identify these to prepare a person-centered approach strategy.

The life expectancy of Koreans is 83.3 years, and old age is prolonging along with the increase in older adult population. Age is one of the most important variables to consider in understanding the life of a prolonged old age [27]. Recognizing older adults over 65 years as a homogeneous group ignores the differences that exist within the older adult group [27], and it is also important to consider various characteristics by subdividing old age to prepare policies for these older adults. In general, old age is divided into older adults in youngest-old adults (ages 65 to 74 years) and old-old adults (75 years or older). In Korea, youngest-old adults make up 8.9% of the total population, which is higher than old-old adults (6.7%) [28]. Previous studies have reported that the lives and experiences of youngest-old adults and old-old adults are different [29,30], and the characteristics related to suicide and suicide ideation differ between youngest-old adults and old-old adults; thus, an approach tailored to age group is required [29,31]. Notably, mental health may deteriorate for youngest-old adults and lead to suicidal ideation. Individuals begin to experience a lack of economic support and role loss due to weakened social support, such as retirement, infrequent social activity, and declining physical functioning [29,30,31,32]. Thus, this study aims to identify factors related to suicidal ideation among youngest-old adults, utilizing data from the National Health and Nutrition Examination Survey (a large-scale national survey in South Korea) and using a decision tree analysis to predict the specific combinations of characteristics among youngest-old adults at high risk for suicide. The specific objectives of this study are described below.

1. To identify factors related to suicidal ideation in young older adults in South Korea.
2. To predict the specific complex characteristics of individuals at high risk to predict suicidal ideation in the young older adults in South Korea.

## 2. Materials and Methods

This study is a secondary analysis of data from Year 1 (2019) of Korea’s Eighth National Health and Nutrition Examination Survey. This survey was approved by the Institutional Review Board (2018-01-03-C-A) of the Korea Disease Control and Prevention Agency (KCDA), and the de-identified data can be downloaded from the agency’s website [33].

### 2.1. Sample and Background

The Korean National Health and Nutrition Examination Survey [33] collects various health-related information, including health level and behavior, nutrition and food intake, and prevalence of chronic diseases. Furthermore, the data have been used in many studies on health promotion, disease prevention, and developing health policy and programs [16,34,35,36]. The survey’s target population is individuals over the age of one residing in South Korea, and it employs two-stage stratified cluster sampling. A total of 8110 participated in the National Health and Nutrition Examination Survey in 2019. Among those included, there were 1018 youngest-older adults, and we removed missing data from the suicidal ideation variable (*n* = 48). Then, this study analyzed a sample of 970 South Korean youngest-old adults (65 to 74 years) [37].

### 2.2. Measurements

#### 2.2.1. Socioeconomic Characteristics

The socioeconomic characteristics assessed in the survey included gender, education level, residential location, employment status, personal income level, family living situation, and spousal status. Gender was divided into male and female, education level was divided into high school graduate or lower and college graduate or higher, and the residential location was divided into “dong” (as urban, lived in a city) and “eup/myeon” (as rural, lived in a town or township). For employment status, “Yes” and “No” were assigned to the employed and the unemployed or economically inactive populations, respectively, and individual income level was divided into quartiles (high, middle-high, middle-low, and low) [33]. For family living situations, “Yes” and “No” indicated respondents living with their family and those who were not, respectively. Furthermore, “Yes” and “No” were used to indicate respondents with a spouse and those without one.

#### 2.2.2. Health-Related Characteristics

Items assessing health-related characteristics included smoking, drinking, sleep, health examination, obesity, hypertension, diabetes, arthritis, physical activity, restricted activity, depression, stress, perceived health, and health-related quality of life. For smoking and drinking, “Yes” and “No” were used to indicate experience or lack thereof, and sleep time duration was divided into categories of 7–8 h (recommend), 5–6 h or 9 h or less (appropriate), and less than 5 h or more than 9 h (inappropriate) [38]. For the health examination, “Yes” and “No” indicated those who had a health checkup in the past two years and those who had not, respectively. For obesity, those with a body mass index of 25 kg/m^2^ or more were considered “obese”, while those with a body mass index less than 25 kg/m^2^ were considered “non-obese”. Hypertension was evaluated according to three stages: normal (not corresponding to hypertension or pre-hypertension stages with systolic blood pressure (SBP) < 120 mmHg and diastolic blood pressure (DBP) < 80 mmHg), pre-hypertension (not corresponding to a hypertension stage with 120 mmHg ≤ SBP < 140 mmHg and 80 mmHg ≤ DBP < 90 mmHg), and hypertension (SBP ≥ 140 mmHg or DBP ≥ 90 mmHg or cases in which the respondent had taken any antihypertensive drugs). Diabetes was evaluated according to three stages: normal (not corresponding to diabetes and pre-diabetes stages with a fasting blood sugar level less than 100 mg/dL or a glycated hemoglobin level less than 5.7%), pre-diabetes (not corresponding to diabetes with a fasting blood sugar level of 100–125 mg/dL or a glycated hemoglobin level was 5.7% and 6.4%), and diabetes (a fasting blood sugar level of 126 mg/dL or more, a medical diagnosis, usage of a hypoglycemic agent or insulin injection, or a glycated hemoglobin level of 6.5% or more). For arthritis, “Yes” and “No” were assigned to cases in which a doctor had diagnosed arthritis (osteoarthritis or rheumatoid arthritis) and those in which there was not such a diagnosis, respectively.

For physical activity, “Yes” and “No” were assigned to cases in which respondents had engaged in physical activities for a considerable period each week (2.5 h or more of moderate-intensity physical activity, 1.25 h or more of high-intensity physical activity, or both activities combined) and those in which they had not, respectively. For restricted activity, “Yes” and “No” were assigned to cases in which respondents were currently restricted in their daily lives and social activities due to health problems or physical/mental disabilities and those who were not, respectively. For depression, “Yes” and “No” were assigned to cases in which respondents felt sadness or hopelessness to the extent that it interfered with their daily lives for two or more consecutive weeks over the last year, and those who did not, respectively. Stress was divided into “high” and “low”, depending on the stress level experienced in daily life. Perceived health was operationalized as the subjective evaluation of one’s health on a 5-point scale (1 = Strongly Agree, 5 = Strongly Disagree). This study reversed the scale such that higher scores indicated better-perceived health. The EuroQOL-5D index evaluated health-related quality of life [33], wherein higher weighted scores correspond to a higher quality [39].

#### 2.2.3. Suicidal Ideation

For suicidal ideation, “Yes” and “No” were assigned to cases in which respondents answered “I have” and “I have not”, respectively, to the question “Have you ever seriously considered suicide in the past 12 months?”.

### 2.3. Data Analysis

We conducted the analyses through complex samples analysis by considering weight, stratification, and cluster variables because the Korean National Health and Nutrition Examination Survey was designed with a complex sample. All analyses were performed in SPSS 27.0, setting the significance level at 95%. We calculated frequencies, percentages, means, and standard errors to describe respondents’ socioeconomic and health-related characteristics. The complex samples’ crosstabs test and a general linear model analyzed each factor’s significance for suicidal ideation. Then, logistic regression analysis identified significant socioeconomic and health-related factors related to suicidal ideation (Aim 1). A decision tree analysis predicted the specific complex characteristics of the group at high risk for suicidal ideation (Aim 2). The algorithm was classification and regression tree (CRT); the respective growth limits of the parent node and child node were set to 2% and 1%, and the maximum tree depth was set to 5. Ten-fold cross-validation confirmed the model’s stability, and the risk estimate value was 0.069.

## 3. Results

### 3.1. The Characteristics and Suicidal Ideation among the Subjects

Of the 970 samples that we analyzed, 7.3% (69 participants) responded that they had seriously considered suicide in the past 12 months. The proportion of the subjects in each characteristic is included in Table 1 and Table 2.

### 3.2. Relationship between Socioeconomic Characteristics and Suicidal Ideation

Table 1 shows the relationship between respondents’ socioeconomic characteristics and suicidal ideation. Among socioeconomic characteristics suicidal ideation was significantly related to education level (χ^2^ = 4.75, *p* = 0.031) and personal income level (χ^2^ = 7.28, *p* < 0.001). Suicidal ideation was significantly more common among those with an education level less than or equal to high school graduation and those whose personal income level was “low”.

### 3.3. Relationship between Health-Related Characteristics and Suicidal Ideation

Table 2 displays the relationship between respondents’ health-related characteristics and suicidal ideation. Among health-related characteristics, suicidal ideation was significantly related to restricted activity (χ^2^ = 9.26, *p* = 0.003), depression (χ^2^ = 92.19, *p* < 0.001), perceived stress (χ^2^ = 43.47, *p* < 0.001), perceived health (*t* = 5.18, *p* < 0.001), and health-related quality of life (*t* = 3.73, *p* < 0.001). Suicidal ideation was significantly more common when respondents had experienced activity restriction, depression, and high-level stress. The scores for perceived health and health-related quality of life were lower in the group with a history of suicidal ideation.

### 3.4. Factors Related to Suicidal Ideation

Table 3 presents factors related to suicidal ideation. The regression analysis included the variables significantly correlated between socioeconomic, health-related factors and suicidal ideation in Table 1 and Table 2. Suicidal ideation was related to personal income level, depression, stress, and perceived health. Suicidal ideation became more common as personal income level decreased (OR = 1.48, 95% CI = 1.04–2.12), when respondents had experienced depression that interfered with daily life for two consecutive weeks (OR = 9.28, 95% CI = 4.57–18.84), as perceived stress levels increased (OR = 2.42, 95% CI = 1.11–5.28), and when respondents reported relatively low perceived health (OR = 1.88, 95% CI = 1.23–3.11).

### 3.5. Predicting the Complex Characteristics of Those at High Risk for Suicidal Ideation

**Figure 1:**
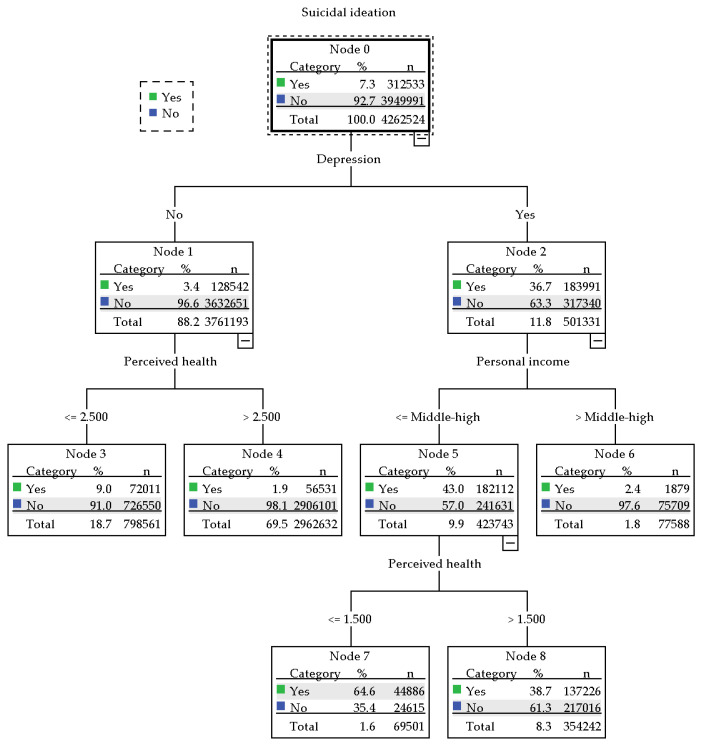
The complex characteristics associated with a high risk for suicidal ideation.

The decision tree analysis predicted the characteristics of the group at high risk for suicidal ideation based on the factors derived from the regression analysis. Suicidal ideation was predicted by combining depression, personal income level, and perceived health (*p* < 0.05); thus, suicidal ideation may occur via the interaction of these three characteristics (64.6%, Node 7; Figure 1). The high-risk group possessed all of the following characteristics: depression, low personal income level (middle-high or less), and low perceived health (1.5 points or less).

## 4. Discussion

Responding to the urgent need worldwide to devise suicide prevention measures tailored to older adults, this study uncovered specific complex characteristics of those at high risk for suicidal ideation. The results provide specific information on this high-risk group of youngest-old adults, which should be prioritized when creating suicide prevention strategies.

First, this study showed through regression analysis that depression, stress, personal income level, and perceived health are significantly related to suicidal ideation in youngest-old adults. Notably, the high correlation between depression and suicidal ideation is consistent with previous studies [10,16,21,35,40,41,42,43]. Paik [29] identified that depression was higher in youngest-old adults with loss of health, economy, and role. Lee et al. [44] reported that 91.7% of older adults with suicidal ideation had been diagnosed with depression. Hu et al. [22] found that depression mediates other variables’ relationships to suicidal ideation among older adults. As the opportunity to participate in social activities decreases due to retirement, youngest-old adults tend to experience loss and depression, resulting in an increased incidence of suicidal ideation [45]. However, not all older adults who have suicidal ideation attempt suicide. Thus, social participation, social support, and social cohesion, which effectively mitigate depression, should be encouraged [45]. Notably, social support can reduce suicidal ideation and depression or stress [45,46].

In the same context, the relatively high rate of suicidal ideation among older adults exposed to high-level stress can be explained based on previous findings that stress is highly correlated with depression [33,47]. Perceived stress is an important factor that increases identification of individuals with higher risk of suicidal ideation among older adults with depression [48]. This result may be attributed to tensions and pressures about an unforeseeable future as individuals experience aging [49], highlighting the need to consider depression and stress management in devising suicide prevention strategies.

Low personal income levels can lead to stress and depression, poverty, loss of social roles, and weakened physical health, increasing rates of suicidal ideation [22,31,32,45,50]. Youngest-old adults may experience suicidal ideation due to various life changes after retirement, including suddenly weakened social support and decreased income [51]. Supporting this reasoning, a recent survey of older adults in South Korea revealed that the main reason for suicidal ideation was economic hardship; 27.7% of these older adults mentioned the issue of living expenses [52], and their annual gross income was proportional to their education level [45,53]. The regression analysis in this study did not show economic hardship as a significant factor, which is consistent with the results of the univariate analysis that demonstrated that suicidal ideation was more common among those with less education (high school graduates or lower). Additionally, many recent surveys have confirmed that economic hardship is a significant predictor of suicidal ideation among older adults [16,32,45], which has important implications for identifying subgroups of youngest-old adults at high risk for suicide [50].

This study revealed that suicidal ideation was more common among those with low perceived health. Older adults occasionally experience difficulties in daily life, leading to loss of usual roles and social contact [54]. These results are consistent with those of a previous study [55] that reported that older adults who perceived their physical health as “bad”, including having activities of daily living (ADL) disorders, showed significantly higher rates of suicidal ideation compared to those who did not [34,56]. Restrictions on social life among older adults in the local community induce social isolation and loneliness and affect depression, increasing suicidal ideation [26]. Thus, management strategies organizing timely visits and establishing a support network using local community resources are crucial for older adults living alone or socially inactive [22,57].

In this study, to prepare a person-centered prevention strategy for suicidal ideation, we identified a high-risk group by combining related factors on the suicidal ideation derived in regression analysis. The decision tree analysis determined that combining the following three characteristics predicted a higher risk for suicidal ideation among youngest-old adults: depression, low income (moderate-high or less), and low perceived health (cutoff point = 1.5 points). Additionally, there is an interaction among physical, psychological, and socioeconomic factors [32,58]. Therefore, it necessary to classify youngest-old adults exhibiting all three characteristics into a high-risk group that should be prioritized when formulating suicide prevention strategies. Suicidal ideation is a strong predictor of suicide attempts [22], and this association is an essential consideration in suicide prevention. Thus, this study provides important insights for identifying high-risk groups among youngest-old adults and has implications for establishing targeted suicide prevention strategies. The findings could help lower the suicide rate among older adults in South Korea and enhance healthcare workers’ ability to discover and manage related issues.

This study has several limitations. First, the data originated from a self-report questionnaire, creating the possibility of subjective bias in responses. Second, since this study is cross-sectional, it cannot interpret the factors related to suicidal thoughts as causal relationships. Therefore, future studies could consider using longitudinal analysis to causally identify and interpret the influence factors. Third, suicidal ideation among youngest-old adults may not necessarily lead to suicide attempts. Fourth, the secondary data utilized in this study may have limitations in identifying all factors related to suicidal ideation among youngest-old adults, especially a history of physical and mental illness, which was not objectively measured. Fifth, this study’s results differed from previous studies [16,59] exploring factors influencing suicidal ideation in older adults age 65 years or older based on the same survey data; thus, future research should consider a variety of respondents, such as other respondent age groups.

## 5. Conclusions

This study identified the specific complex characteristics of youngest-old adults at high risk for suicidal ideation in South Korea, with the world’s highest suicide rate and a rapidly aging population. This high-risk group exhibited depression, low personal income, and low perceived health. The results suggest relevant criteria for selecting target demographics in establishing suicide prevention strategies for older adults.

Furthermore, this study has two important implications. First, target selection and customized interventions should be implemented based on vulnerable individuals’ characteristics to prevent suicide among older adults in South Korea. Second, although this study revealed that physical, psychological, and socioeconomic factors are jointly related to suicidal ideation among youngest-old adults, future research should expand the model to include other potentially influential factors.

## Figures and Tables

**Table 1:** The socioeconomic characteristics associated with suicidal ideation among the subjects.

Variables		Total (*N* = 970)	Suicidal Ideation		
			No (*n* = 901)	Yes (*n* = 69)	Rao–Scott χ^2^
		*n* (%)	*n* (%)	*n* (%)	
Gender	Male	427 (47.7)	399 (93.3)	28 (6.7)	0.39 (0.535)
	Female	543 (52.3)	502 (92.1)	41 (7.9)	
Education level	≤High school	817 (87.0)	757 (92.7)	60 (7.3)	4.75 (0.031)
	University	106 (13.0)	104 (98.3)	2 (1.7)	
Living location	eup/myeon	723 (77.5)	670 (92.4)	53 (7.6)	0.37 (0.544)
	dong	247 (22.5)	231 (93.7)	16 (6.3)	
Economic activity	Yes	388 (42.7)	365 (94.9)	23 (5.1)	1.92 (0.168)
	No	538 (57.3)	497 (92.0)	41 (8.0)	
Personal income	High	240 (26.7)	235 (98.1)	5 (1.9)	7.28 (<0.001)
	Middle-high	235 (24.1)	225 (95.7)	10 (4.3)	
	Middle-low	252 (24.5)	228 (89.3)	24 (10.7)	
	Low	239 (24.6)	209 (86.9)	30 (13.1)	
Family living situation	Yes	792 (84.7)	737 (92.9)	55 (7.1)	0.31 (0.581)
	No	178 (15.3)	164 (91.6)	14 (8.4)	
Presence of spouse	Yes	735 (78.1)	683 (93.2)	52 (6.8)	1.66 (0.200)
	No	226 (21.9)	209 (90.4)	17 (9.6)	

N: unweighted; %: weighted; Rao–Scott χ^2^: a correlation between socioeconomic characteristics and suicidal ideation.

**Table 2:** The health-related characteristics associated with suicidal ideation among the subjects.

Variables		Total (*N* = 970)	Suicidal Ideation		
			No (*n* = 901)	Yes (*n* = 69)	Rao–Scott χ^2^ or *t* (*p*)
		*n* (%) or M ± SE	*n* (%) or M ± SE	*n* (%) or M ± SE	
Smoking	Yes	396 (43.6)	367 (91.5)	29 (8.5)	0.93 (0.337)
	No	574 (56.4)	534 (93.6)	40 (6.4)	
Drinking	Yes	787 (83.2)	729 (92.2)	58 (7.8)	1.77 (0.186)
	No	183 (16.8)	172 (95.1)	11 (4.9)	
Sleep time (Weekday, hours)	7–8	428 (44.3)	402 (93.6)	26 (6.4)	1.33 (0.267)
	5–6, 9	439 (45.3)	411 (92.8)	28 (7.2)	
	<5 or >9	103 (10.4)	88 (88.4)	15 (11.6)	
Sleep time (Weekend, hours)	7–8	449 (46.2)	425 (94.3)	24 (5.7)	1.70 (0.184)
	5–6, 9	412 (42.1)	382 (91.9)	30 (8.1)	
	<5 or >9	109 (11.8)	94 (89.1)	15 (10.9)	
Health examination	Yes	720 (77.8)	673 (93.7)	47 (6.3)	1.01 (0.316)
	No	205 (22.2)	188 (91.5)	17 (8.5)	
Obesity	Obese	342 (35.1)	316 (92.4)	26 (7.6)	0.04 (0.845)
	Non-obese	625 (64.9)	582 (92.8)	43 (7.2)	
Blood pressure	Normal	155 (17.0)	140 (91.2)	15 (8.8)	0.43 (0.649)
	Pre-hypertension	217 (22.2)	207 (94.1)	10 (5.9)	
	Hypertension	597 (60.8)	553 (92.5)	44 (7.5)	
Glucose	Normal	188 (20.9)	176 (94.9)	12 (5.1)	1.09 (0.335)
	Pre-diabetes	443 (51.2)	415 (94.3)	28 (5.7)	
	Diabetes	264 (28.0)	244 (91.3)	20 (8.7)	
Arthritis	Yes	277 (28.7)	256 (93.6)	21 (6.4)	0.09 (0.771)
	No	649 (71.3)	606 (93.0)	43 (7.0)	
Physical activity	Yes	348 (38.1)	329 (94.5)	19 (5.5)	0.90 (0.345)
	No	576 (61.9)	531 (92.4)	45 (7.6)	
Restricted activity	Yes	124 (11.6)	107 (86.6)	17 (13.4)	9.26 (0.003)
	No	802 (88.4)	755 (94.1)	47 (5.9)	
Depression	Yes	129 (11.8)	84 (63.3)	45 (36.7)	92.19 (<0.001)
	No	841 (88.2)	817 (96.6)	24 (3.4)	
Perceived Stress	High	164 (16.1)	127(77.2)	37 (22.8)	43.47 (<0.001)
	Low	806 (83.9)	774 (95.6)	32 (4.4)	
Perceived health		3.00 ± 0.03	3.05 ± 0.03	2.28 ± 0.13	5.18 (<0.001)
HRQoL		0.92 ± 0.01	0.92 ± 0.01	0.83 ± 0.03	3.73 (<0.001)

HRQoL = health-related of life; M = Mean; S = Standard error; N: unweighted; %: weighted; Rao–Scott χ^2^: a correlation between socioeconomic characteristics and suicidal ideation.

**Table 3:** Factors related to the suicidal ideation (*N* = 970).

Variables		Suicidal Ideation		
		OR	95% CI	*p*
Education level	≤High school	1.97	0.33–11.64	0.451
	University	1		
Personal income (Ref: High)		1.48	1.04–2.12	0.030
Restricted activity	Yes	0.78	0.37–1.62	0.499
	No	1		
Depression	Yes	9.28	4.57–18.84	<0.001
	No	1		
Perceived stress	High	2.42	1.11–5.28	0.026
	Low	1		
Perceived health (Ref: Very good)		1.88	1.23–3.11	0.016
HRQoL		0.99	0.79–1.24	0.927

HRQoL = health-related of life; Ref = reference value; OR = odds ratio; CI = confidence interval.

## Data Availability

Researchers who want to use microdata and analytic guidelines can be downloaded from the KCDA website (https://knhanes.kdca.go.kr/knhanes/main.do (accessed on 16 April 2021)) in Korean.
